# Materiality of Memorialisation: Mapping Migrant Women's Landmarks in Europe

**DOI:** 10.12688/openreseurope.18433.1

**Published:** 2024-10-25

**Authors:** Bénédicte Miyamoto, Maija Ojala-Fulwood, Veronika Čapská, Fiona Eva Bakas, Igor Lyman, María Amor Barros-del Río, Maria Bostenaru Dan, Alba Comino, Pirita Frigren, Victoria Konstantinova, Heidi Martins, Lívia Prosinger, Pauliina Räsänen, Biljana Ristovska-Josifovska, Marie Ruiz

**Affiliations:** 1 Université Sorbonne Nouvelle, Paris, France; 2 Tampere University, Tampere, Finland; 3 Institute of Philosophy, Czech Academy of Sciences, Prague, Czech Republic; 4 Universidade Lusófona, Lisbon, Portugal; 5 Berdyansk State Pedagogical University, Zaporizhzhia, Ukraine; 6 University of Burgos, Burgos, Spain; 7 Ion Mincu University of Architecture and Urban Planning, Bucharest, Romania; 8 Universidade Nova de Lisboa, Lisbon, Portugal; 9 University of Turku, Pori, Finland; 10 Centre de Documentation sur les Migrations Humaines, Dudelange, Luxembourg; 11 Institute of Political History, Budapest, Hungary; 12 University of Turku, Turku, Finland; 13 Institute of National History, Skopje, North Macedonia; 14 Université de Picardie Jules Verne, Amiens, France

**Keywords:** gender inequality, migration, toponomy, cultural memory, memory politics, critical place-naming

## Abstract

This article investigates the memorialization of migrant women across transcultural landscapes, and analyses results from the Register of Migrant Women Landmarks in Europe (hereinafter RMWLE), central to the European Cooperation in Science and Technology (COST) action project “Women on the Move” (CA19112 – WEMov). It serves as reference for subsequent research based on data from this Register, for which data collection is continuing. The RMWLE registers toponyms, such as monuments, plaques, streets and other infrastructures named after women with a significant history of migration. It honours aspects rarely prioritized in memorialisation agendas, which are skewed towards men’s stories, and towards the more linear biographies of sedentary figures whose European, national, and regional memorialisation have remained uncomplicated by migration.

This Deep Data study reveals recurring patterns at the level of Europe in the memorialisation of these women migrants. The diversity of stories, the richness and the prominence of landmarks devoted to men compared to women is a subject well-covered in memorialisation studies. This unbalance is compounded by the data from our register which shows landmarks on women migrants that are sometime tokenized, often marginalized, and which reproduce the bias towards nurture and care that have besieged the memorialisation of women in general. It further shows that the memorialisation process and the political and cultural mechanisms of official celebration often work against the recognition of cross-border careers and stories.

The intersectionality of the project, highlighting both gender and migration, uncovers a political landscape of toponyms – and we reflect on how this register can help combat cultural prejudice by recovering migration episodes. The RMWLE helps us reflect on the defining impact of migration episodes, a reality rarely underlined in the biographies of famous women. This article favours a storytelling approach, to counter dominant cultural narratives and knowledge practices.

## Plain language summary

This article is interested in how famous women who have migrated are remembered across European countries in ways that makes their name public and visible, be it through street names, plaques, statues or monuments for example. This article explains the methods, results and difficulties that went into creating the Register of Migrant Women Landmarks in Europe (hereinafter RMWLE), central to the European Cooperation in Science and Technology (COST) action project “Women on the Move” (CA19112 – WEMov). Women have long achieved public fame and have built important careers even while migrating, and often thanks to their migration. This article investigates how and where these figures are honoured and given pride of place in European nations, and reflects on their marginal or tokenized presence in European cities. We compare these women migrant landmarks to monuments built for their male counterparts, we trace how migrant women are often bypassed in favour of national figures that have not migrated, and we advocate for a more diverse representation of meaningful female figures to celebrate in European cities.

## Introduction

The Register of Migrant Women Landmarks in Europe (hereinafter RMWLE), central to the European Cooperation in Science and Technology (COST) action project “Women on the Move” (CA19112 – WEMov) 2020–2024, registered not only odonyms, (street names) but a vast array of urban toponyms (from monuments to such varied urban infrastructure as nurseries and hospitals). The project is ongoing, but a dataset collected from September 2020 to July 2023 is accessible ( Bakas *et al.*, 2023). This toponomy of women with a significant history of migration honours aspects rarely prioritized in memorialisation agendas. We underline how women migrants’ achievements are overshadowed in European, national, and regional cultural celebrations compared to men’s achievements and compared to figures whose biographies have a more linear narrative, uncomplicated by migration. The diversity of stories, the abundance and the prominence of landmarks devoted to men compared to women is a subject well-covered in memorialisation studies. This unbalance is compounded by the data from our register which shows landmarks on women migrants that are sometime tokenized, often marginalized, and which reproduce a gendered bias towards nurture and care that have besieged the memorialisation of women in general. It further shows that cultural memory relies on a definition of collective identity that is often subsumed in national identities, with toponyms often forging a trivialized, habitual patriotism ( Rutkowski, 2023). Therefore, the memorialisation process and the political and cultural mechanisms of official memorialisation have often worked against the recognition of cross-border careers and stories. Less supported by institutions who tend to curate local, regional or national figures, and often put aside in the focused research of experts and historians who still tend to be specialist of an area as well as a period, these cross-border celebrations remain rare. The dataset delves into the legacy of migrant women, a legacy that is shared in two or more countries and are often politically and culturally contested. The resulting article benefits from the rise of transcultural studies intending to interrogate memory between and beyond borders and participates in the critical turn in place-naming studies ( Bond & Rapson, 2014; Rose-Redwood *et al.*, 2010).

Recovering the toponomy of women migrant in the European landscape has uncovered many questions. Firstly, what's in a name? The "politics of place" has been an active - and activist – research topic in the last decades ( Cresswell, 2004). It has renewed our appreciation of how much public landmarks, which are often taken for granted or fading away in the fabric of our everyday commute through the city, are in fact negotiated and contested ( Berg & Vuolteenaho, 2016). The cultural debate has emphasized that memorialization of public figures has an impact that is more than just cosmetic or symbolic, and that it influences the cultural debate, shaping the identity of heritage at a local and national level, in ways that have tended to reproduce a dominant narrative of systemic injustice ( Clerval *et al.*, 2015).

Secondly, the register interrogates why and which landmarks matter. This article explores the visibility of women through toponomy, and their minoritization. We discuss the cultural politics and the politics of memory behind the stories celebrated in the urban landscape, keeping in mind that “how people construct the past through a process of appropriation and contestation” also interrogates the very act of memorialisation that this register is built on ( Confino, 1997, 1403). By making space in this article for these migrant women’s life stories, we are inspired by the recent push to shape history through renewed narratives ( Andrews, 2007; Maurantonio, 2014) and through a narrative framework of resistance ( Smith, 2017). Ultimately, we hope to foster reflexivity from the audience when confronted with interacting and interlocking stories of female working and migrating life experiences ( Gherardi & Poggio, 2009).

Thirdly, the register discloses a class bias in memorialisation with an overwhelming celebration of women from the upper-classes, together with a persistent gender-coding, especially in relation to professions and the perception of traditional feminine roles in the fields of care and education. Looking at the gender balance of street names and statues helps put the national or city narratives to right and eschew former patriarchal or exclusive narratives. Naming a street or commissioning a statue is a political act, inscribing an authorized version of history into ordinary settings of everyday life ( Azaryahu, 1996; Calotă, 2010). That authorized version is evidently not consensual. The public space is the space of memory politics, the space of decision making and negotiation. As the art historian Milena Bartlová argues, “visual, or rather material images can be – and very often are – a visible and tangible embodiment of power relations and that the erection or removal of a public sculpture is as political an act as there can be” ( Bartlová, 2021, 134). In this sense, the RMWLE stands as a political act in its gathering of women migrant landmarks and its highlighting of their heritage.

The selected landmarks and migrant trajectories in the RMWLE feature cross-community or cross-cultural migration. They show both typical and exceptional forms of mobility and present women of different age, profession, social status and migration status. This intersectionality of the project and the dataset highlights not only the richness of these landmarks and their value for scholarship but also the wide spectrum of migrant women and their contribution to society.

## Method and data collection

The methodology used in this work combines the "Qualitative Content Analysis" and "Case Study" methods based on a dataset. The "Qualitative Content Analysis" method focuses on identifying patterns, themes, and categories within the *Register of Migrant Women Landmarks in Europe* dataset ^1^. This approach enables an analysis of how migrant women are represented and referred to in various European urban settings. The data collection, which took place in different European regions and languages, relied on the linguistic expertise of the COST WEMov network members, and their qualitative in-person knowledge of local topographies. Meanwhile, through the coding of references to migrant women in different European contexts, the "Case Study" method allows for an in-depth examination of specific scenarios, such as the treatment of migrant women in a particular European country or professional sector.

In Humanities and Social Sciences research, thorough documentation —including case studies and guidelines— is essential to ensure both the reproducibility of the research and the usability of the resulting resources ( Middle, 2023). The collection of data is an ongoing process which started in September 2020 and the dataset to which this article refers presents the data collection as of July 2023. The available empirical material of migrant women landmarks “in situ” is subject to change as the memorial landscape evolves, and our research has been deliberately conceived as open-ended and open access. The WEMov project dataset adheres to the FAIR principles, making it easy to find and accessible via this NAKALA repository. The data are interoperable and reusable in other research thanks to its CC-BY-4.0 license. Additionally, we have made efforts to align the dataset with the Checklist to Publish Collections as Data in GLAM institutions ( Candela *et al.*, 2023). Consequently, the proposed methodology, in combination with the dataset, ensures the reproducibility of the results.

The dataset falls under the concept of Deep Data versus Big Data, as we work with structured data digitally curated through a complex review system. The term Deep Data refers to a dataset that is not very big, but semantically rich, and provides contextually wealthy information intended to provide a rewarding user experience ( Štular & Belak, 2022). The main aim of the RMWLE is to provide the variables for each occurrence of migrant women’s landmarks in Europe, in columns comprising landmark categories, geo-coordinates, address, country, short description, the identified woman’s occupation as well as its ESCO category, and her standardized name and biographical dates.

The processing and creating of this type of data is time-consuming because the annotation is heavily dependent on human analysis. The collection of data was based on voluntary work of scholars and students from over 40 different European countries. As we worked on the database, we were faced with the following challenges: who is counted as a migrant woman and thus should be included in the RMWLE? What trajectories count as migratory? How far, how long, and how frequent did mobility have to be to count as transnational cross-borders migration? We acknowledged different degrees of intensity and frequency in the migration and re-migration episodes of these women, choosing to look for significant societal roles and functions involving both their home and host societies, according to current definitions of a transnational social field ( Boccagni, 2012; Levitt & Glick-Schiller, 2004). Indeed, a strict national border criterion cannot not be used consistently, and current definitions of the transnational draw on the concept of cross-community migration ( Manning, 2006). Given the long historical period covered and the upheavals to which borders have been subjected, our project accounts for various forms of geographical mobilities which represent, in our actor-centred understanding of migrant biographies, significant changes of cultural and political spaces.

All landmarks in the RMWLE therefore refer to a significant episode of migration in one or more women’s history, understood as cross-community or cross-cultural migration. They show both typical and exceptional forms of mobility and present women of different age, profession, and social and migratory status. The gender of the women memorialized in statues, monuments, or plaques across Europe, and celebrated with schools, museums or hospitals named after them is often central to the acts of memorialisation. That their biographies frequently uncover varied and diverse stories of migrations is less well known, rarely commented upon when such monuments are erected and remains very much in the background of feminist activism to increase the number, quality and inventiveness of female landmarks across Europe. Reclaiming the migratory episodes of these women’s careers and trajectories is central to the RMWLE’s goals, as it underlines that “the analytical precision necessitates the term migrant to be used more systematically, precisely because we must put to rest the idea that definitions are only operative if they gauge how voluntary or self-willed the act of migration is, has been or will continue to be” ( Miyamoto & Ruiz, 2021, 11). We hope that this RMWLE and its labour of biographical and mnemonic care will redress this unbalance and fuel research on the intersection of gender and migration.

Another challenge regarding what landmarks should be harvested was the question of what or who the landmark represented. We choose to harvest data on migrant woman memorialised as embodied and not just allegorical. Numerous previous studies about women represented in public art have harvested such allegorical representation as denoting female presence in the city landscape nonetheless, but our selection criteria that cross reference womanhood and the experience of migration in the biography of the woman represented made this category of monument irrelevant to the register. Not only are allegorical representation devoid of biography, but they often erase women’s lived experiences and enhance stereotypical features of womanhood. This project favours a storytelling approach, to counter dominant cultural narratives and knowledge practices, and for effective storytelling, commemorative place naming had to have taken place for the landmark to be registered. In some cases, landmarks with family, married couple or a collective were included, but only if the migrant women had played a significant role and were specifically identified by name, by profession or by clear representation. Some of the landmarks we accepted are unnamed or generic, but still clearly defined as a migrant woman or women. In general, landmarks were not accepted unless they were dedicated or named after the woman migrant in a significant act of commemoration. The act needed to be mnemonic, intent on leaving the woman’s name or profession as an imprint on the social landscape.

Geared towards providing a cartographic visualisation of these landmark, the data collection strove for geographical balance while harvesting the data. The effort of collecting this data is still ongoing, but some areas are better represented as the RMWLE stands due the nature of data collection. The results are not exhaustive enough to enable us to make quantitative comparisons by national categories yet, but some patterns are already pointing to which European countries have traditionally or more recently awarded space to the commemoration of migrant women. These are consistent with similar research conducted on European toponyms ( Corona & Ferrari, 2023; Ouali *et al.*, 2021). Cultural differences in place-naming do not always pertain to nationalistic or male-centred practices, however. Commemorative place-naming in certain Slavic European countries, for example, is less honorific and person-based than in others, favouring the memorialisation of dates, events, or political values instead, especially throughout the urban expansion of the twentieth century ( Jaroslav, 2011).

What about the categories of landmarks that emerged from our data collection? At 1000 entries strong, it is possible to refine and discuss the categories of landmarks in this project. The project's main goal was to harvest landmarks that heightened the visibility in the landscape of migrant women, and which recognized more diverse achievements. We therefore quickly broadened the register of classic commemorative landmarks of plaques, monuments and statues to include all sorts of toponyms (the proper names of places, also known as place name or geographic names), oeconym, (the proper name of a house or any other residential building), astionims (proper names of cities and towns), as well as urbanonyms (the proper names of urban elements such as streets, squares etc. in settlements) which include agoronyms (proper names of squares and marketplaces) and hodonyms (proper names of streets and roads). This enabled a fuller register of the political landscape in the RMWLE which now includes elements such as tombs, lighthouses, and waterfalls, among others. Finally, the harvesting was classified in sixteen different categories of commemorative landmarks: burial (cemetery, mausoleum, tomb...); cultural venue (cinema, library, museum, theatre...) educational venue (school, university...); former residence; green space (garden, promenade, square...); housing (flat, council homes...); institute (foundation, research centre...); monument (bust, statue...); medical venue (hospital, retirement home...); natural site (bay, lake, waterfall...); plaques (murals, stepping stones); religious venues (church, mosque, synagogue...); sports venue (stadium, swimming pool); street (avenue, boulevard, road...); temporary installation; and transport infrastructure (airport, bridge, bus stop, station...).

Normalization of the labour information was necessary in order to deal with coherent sets of data – a specifically difficult harmonization in the case of a corpus stretched over a long historical period and extended cross-borders. The RMWLE was therefore sorted according to the European framework of European Skills, Competences, Qualifications and Occupations (ESCO). This classification identifies and categorises the professional components relevant for the EU labour market. Although this framework seems oriented towards data from the twenty-first century, the ESCO is in fact convenient to use for historicised data since it is based on skills and competences. Framework based on qualification- and occupation are difficult to adapt to historicised data, especially with a gender under scrutiny that often was not granted clear occupational titles to go with the labour they provided or was barred from attaining specific qualifications. The ESCO helps us describe the women in our data in terms of responsibility and autonomy. This can help us understand the position of these women in society and their influence, impact and leverage (which is the structural incrementation that is used to go from 0 to 9 in the framework) thanks to a competence-based approach, which ultimately supports a coherent study of work and cross-border mobility, because with the ESCO classification, "competence means the proven ability to use knowledge, skills and personal, social and/or methodological abilities, in work or study situations and in professional and personal development” ( European Commission, 2018).

How did we collect and harvest this landmark data? Our network linking researchers over 40 European countries mined a corpus of academic bibliographies on urban surveys, infrastructure indexes, and lists of toponymic and gender issues; we sifted through public information notices such as municipal and town hall minutes, local newspapers articles covering toponym news. We used national registers and inventories of cadastral databases (with country specific geoportals such as the Romanian ANCPI geoportal, the Walloon Inventaire Centralisé des Adresses et des Rues (ICAR) or the French IGN Géoportail) or street names advocacy projects (such as the Romanian project at strazicurenume.ro). We then effected a selection by identifying significant migration episodes in the women-based toponyms of the corpus. These results were then cross-referenced for the appearance of these women’s names in geo-localisation web services (such as OpenStreet Map™, Google Maps™ or MapQuest™) which included a wider range of toponyms. Landmarks identified were entered by a first contributor to a Google form and were then checked for conformity of data by a separate contributor before it was included on the landmarks register spreadsheet.

This demanded extensive interdisciplinary cooperation within the COST network and allowed for outcome-oriented student workshops. The Excel spreadsheet dataset was completed on July 2023 and is available on NAKALA. Also, a Data Note is available to give a brief description of the dataset, including details of why and how it was created, and to promote the reuse of the data for research purposes ( Ojala-Fulwood *et al.*, 2024). Finally, an interactive map on landmarks, which is intended as both a visualisation of our research and an educational interactive tool, is available on the Map of Women Migrants' Landmarks on the website. ^2^

## Results

### 1) Memorializing migrant women in Europe remains skewed towards the elite

Naming landmarks is an act that necessitates power and decision leverage, and therefore even more susceptible to be a space of systemic patriarchy and mainstream. The ability to control the meaning of place is an aspect of power and memory politics. The European Data Journalism Network project “Mapping Diversity” has evidenced that on average 91% of the streets named after individuals are dedicated to men ( https://mappingdiversity.eu/). Monuments for women, especially migrant women are also conspicuous by their absence. Citizens have gained a heightened sense of this imbalance in the recent decades. As the sculptor Anna Franziska Schwarzbach reflected upon the unveiling of her statue of Lise Meitner in Berlin, this awareness justifies the statue’s oversized pedestal: "Outstanding women are rarely put on a pedestal. How difficult it must have been for a woman to work in science, how even more difficult to be respected. That gave me the idea to make the pedestal as wide as possible, as a way to commemorate the many 'non-pedestalled” ( Schwarzbach, 2014). Calls for more space devoted to usually non-pedestalled migrant women figures have risen, as perception of the socially constructed nature of the urban space around us has spread ( Entrikin, 1991).

Indeed, if naming places has often reinforced “claims of national ownership, state power, and masculine control," more recently “attempts to rename (and in doing so, reclaim) places are implicated in the discursive politics of people and place.” ( Berg & Kearns, 1996). The addition in 2002 of a migrant female figure, Marie Dentière (c. 1495–1561), to the ten male statues of the Reformation Wall in Geneva did little however to appease criticism. Although she played an active role in Genevan religion and politics, sustained by prominent public preaching, Dentière’s role was minimized by the male-centred archival curation of Geneva’s religious past. It is increasingly argued that cultural landscapes gain in transcultural dynamics and strengthen the perspective of alterity if they include not only the perspective of gender but also that of migratory trajectories ( Čapská, 2025). Yet, the RMWLE shows that historical significance and visibility is rarely granted to those that are or have been minoritized. The fact that Marie Dentière’s plaque, rather than a statue, is neither central nor self-standing, placed as it is at the foot of a male statue, continues therefore to cause outrage. The begrudging inclusion proves insufficient to rebalance the monument’s legacy of public memory. Such minimal acknowledgement, with a reduced position of female stories of migration is an unsatisfactory compromise that the RMWLE has uncovered multiple times.

The RMWLE project has sharpened our understanding of the myriad of obstacles encountered by historic and cultural urban projects advocating for more inclusive commemorative practices. Place names can indeed be read as “part of the official memory of a city or a nation, [and as such they act] as evidence of social norms and values at a given time,” and are a “highly political process and a way of symbolically recognising individuals or groups of people. This also means that city maps can be perceived as a documentation of social and political power relations” ( Hintermann & Pilcher, 2015, 291-2). Therefore, initiatives such as the projects Toponomastica Femminile or Merezco Una Calle, have put the spotlight on the absence of women in urban spaces, and promoted a more egalitarian presence of women in the cities.

The RMWLE testifies that a more diverse urban landscape is needed. Nowadays, urban projects largely intend to “strengthen urban storytelling and enhance citizens’ interest in history” ( Hayden, 1996, 228). Officials are faced with a glut of prejudicial public art foregrounding values which are now inconsistent with the avowed public values of the very city, region or nation taking care of their upkeep ( Doss, 2018). Urban planners also recognize the benefits of righting the balance by naming and celebrating recovered and unsung figures. This has been highlighted recently in the Sustainable Development Goals established by the United Nations General Assembly in 2015. The official mission of its eleventh goal seeks to make cities inclusive, safe, resilient and sustainable, hence encompassing the issue of public art’s historically troubled relationship between conservation on the one hand and the fostering of diverse and socially representative public art on the other. Previous reports on the relationship between historic urban areas and cultural diversity and social inclusion have drawn bleak conclusions and underline the invisibility of women in the historic urban landscape. When intersected with migration, the results are even starker. Results of a 2018 World Heritage Convention and UNESCO consultation survey indicate that integration policies for migrants in historic urban areas averaged between 15% to 16% in European states ( UNESCO, 2019, 12–13). The place of migrants, migrant stories and migration biographies is even more underserved by cultural projects rejuvenating historic urban landscapes.

**Figure 1.**
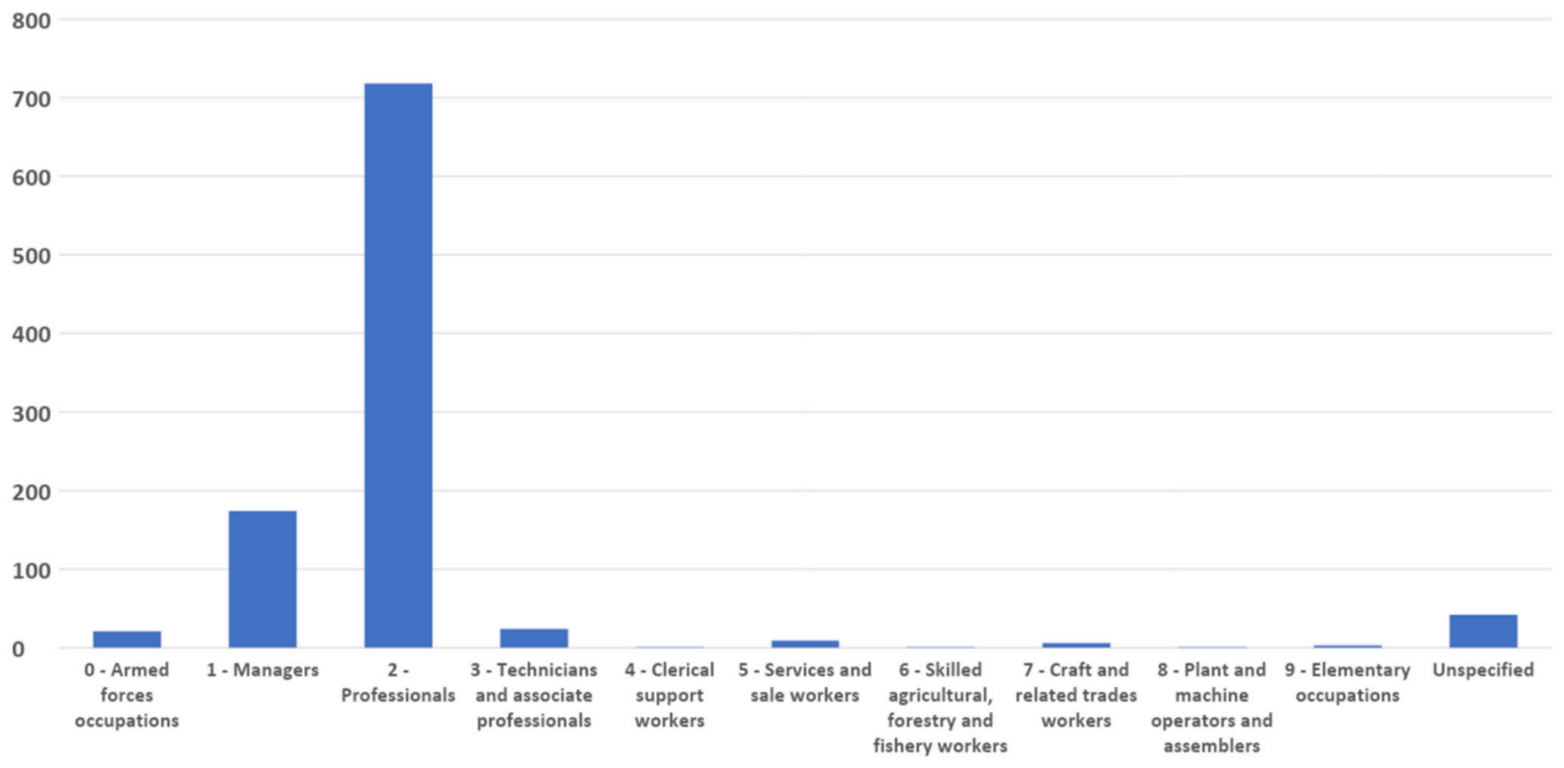
Migrant women’s occupation in the Register of Migrant Women Landmarks. Credit line: Authors. This bar graph compares the occupation of women memorialized by landmarks in Europe, based on 1000 entries of the Register of Migrant Women Landmarks using the European Skills, Competences, Qualifications and Occupations (ESCO) taxonomy. The dataset can be found on NAKALA at https://doi.org/10.34847/nkl.28cf9j6q ( Bakas *et al.*, 2023). Most memorialized women had a professional or managerial career (ESCO 1 to ESCO 3), while women from clerical to elementary occupations (ESCO 4 to ESCO 9) are vastly underrepresented compared to the percentage of women pertaining to these categories, historically and now, in contrast to elite careers.

When being a woman and having an episode of migration is compounded with an underprivileged social background, presence in the RMWLE is even rarer. Low paid occupations rely on sets of skills that are defined by the labour market as low because they are negatively correlated to one’s amount of influence in society and to the extent of decision taking one is granted. In the RMWLE, representations of migrant women from the ESCO Classifications of labour going from Clerical (ESCO classification 4) to Elementary occupations (ESCO occupation 9) are conspicuous by their absence, although governesses, seamstresses, secretaries, factory workers and agricultural workers have long been the main professions of the bulk of migrant women. (See Figure 1). Rarely are landmarks made in the cultural landscape for those migrant women at the bottom of the social ladder, and when they are, they are often represented as collective entities, in the absence of named records or recorded biography. The RMWLE holds only one entry for Clerical support (ESCO category 4), for example. This anonymous fate befalls the “potato girl” represented in the sculpture “Kartoffelpigen” in Fredericks Danemark, which depicts an anonymous girl, with potatoes on her lap and her back shaped like a potato. It was erected in 1984 by Ole Mynster Herold to commemorate the efforts of the potato farming workers, their interaction with the native population and the root crop they introduced to Denmark. Gottfred Eickhoff's bronze sculpture "Roepiger", erected in 1940 likewise shows unnamed "Beet Girls," which form a monument to the agricultural workers from Poland and elsewhere who worked in the beet fields in Denmark from 1892 to 1929, most of whom were women. Memorialisation of these migrant workers places women in public spaces and helps transgress the traditional approach that inscribes women in the private and domestic spheres ( García & Rice, 2023). Regrettably, such efforts of memorialization provide but a partial recovery of their stories: these women are memorialized but remain nameless.

Even though some landmarks in the RMWLE are both nameless and without profession, they are still included because they have stories to tell and are far from allegorical representations. The 1981 *Gli Emigranti* bronze statue by Domenico Ghidoni in Brescia represents an unnamed woman and daughter waiting for departure, sitting on a bench with their luggage as personification and collective representation of migration. Although such statues often recycle the form and composition of "Charity" allegories, they do not choose the female form as aesthetically pleasing to represent a concept but to embody a lived experience and recognize that such women have left too little traces in history to be named. These monuments are therefore an effort of representation and visibility in favour of women migrants – a reconstruction and recovering act of care in the face of historical erasure of such records. Ghidoni’s *Gli Emigranti* uses realism in its portraiture, not idealization – more than a hundred years later, it continues to resonate with migration’s lived experience, and it has been moved from the secluded recess of the museum to the open space of the parco Torri Gemelle in Brescia in 2023.

Collective memorialisation of female migrants can also be linked to war crimes and atrocities. In Helsinki, Finland, the statue of a war evacuee-mother erected in 2015 commemorates Carelian war evacuee-mothers who were displaced during the Second World War. The sculpture of a refugee woman with child at the Waldfriedhof Troisdorf cemetery in Germany memorialises WWII expellees. Such monuments devoid of the precise names and professions of these individuals who have lost their families, and even community, still acknowledge them as victims who deserve to be mourned transnationally and who deserve recognition, reparations and/or public apology by the State ( Hilmar, 2020).

On the other hand, the RMWLE finds an abundance of landmarks that represent migrant women with a history of power and authority, demonstrating competent leadership skills in society, and tagged as Managers (ESCO category 1), or Professionals (ESCO category 2) on the ESCO categories. Rulers, politicians, scientists, writers, and acclaimed creative artists and performers, among other socially influential activities, are registered in abundance. However, a closer look at the descriptions reveals that out of the 174 Manager classification, very little are Commercial (ESCO classification 1-12) Production (ESCO classification 1-13) or Hospitality Managers (ESCO classification 1-14). The bulk of the 174 landmarks of migrant managerial women also represent women’s whose position in life was awarded to them through hereditary power lines. Queens, princesses, and other aristocrats benefited from extensive and lavish public memorialisation, and count for one half of these Managerial landmarks. Furthermore, their migration from one national court to another national court, or elite circle, was a mostly privileged experience of cultural mobility. Leaders of political parties, suffragettes, activists and resistance leaders, frequently from a less privileged background, had a more tenuous grip on power.

In the RMWLE, the largest bulk of representations is Professionals (ESCO classification 2), with 71% of the total landmarks. A little less than 50% of these professionals are classified as authors, journalists and linguists (ESCO classification 264), which were careers traditionally more open to women, and often doubled with the profession of translator, a professional skill directly derived from their migration history. The remaining professionals represented are of three sorts: religious professionals, such as nuns (ESCO classification 2636) and teachers (ESCO classification 23) make up for 10% of the landmarks. These were traditional career paths for intellectually-minded women, as well as professional options which patriarchal structures were keener to memorialise than other less gender-coded activities. Science and engineer professionals (ESCO classification 21) make up around 10% of the total landmarks and their memorialisation has recently been accelerated by campaigns about women in STEM. Finally, legal, social and especially cultural professionals (ESCO classification 26) make up another 10%. These cultural professionals’ landmarks celebrate creative artists such as painters and other visual artists (ESCO classification 2651), but more generally performing artists such as opera singers and dancers (ESCO classification 2652), a testimony to the gendered celebrity culture of late nineteenth-century Europe which enabled a few performing individuals to achieve fame outside the home ( Berlanstein, 2004). These operatic and stage careers were often seen as exceptional, based on the concepts of special abilities and talent, while international contracts and related mobility in these careers were viewed as successful. However, here again, social class considerations intervene in the memorialisation. Few landmarks commemorate female circus performers or female magicians, who travelled widely across Northern European countries, including Finland, Sweden, Norway, and Denmark, during the Golden Age of circus and magic arts in the 1880s–1930s. Unfortunately, a tangible part of history—women entertainers in popular entertainment—has thus been forgotten and marginalized. One exception is the slack-wire sensation Elvira Madigan (1867–1889), with several landmarks, streets, and even a museum dedicated to her. However, the reasons for her memorialisation are here again part of a celebrity culture that romanticized tragic women figures rather than celebrated models of entrepreneurial success. A poignant case is internationally successful circus artist and acrobat Elvira Bono (1893–1973) who lacks commemoration, and her tombstone makes no reference at all to her artistic achievements. To integrate into Finnish society, Bono had changed her last name to Sormunen after her third husband. Her acquired name, "Elvi Sormunen," is engraved on to a modest tombstone at Rovaniemi cemetery in Lapland, Finland, making it difficult to recognize her. Her career in the circus remains uncelebrated in the histories of entertainements and national history ( Räsänen, 2019). Due to her gender, profession, and immigrant status, she belonged neither here nor there.

**Figure 2.**
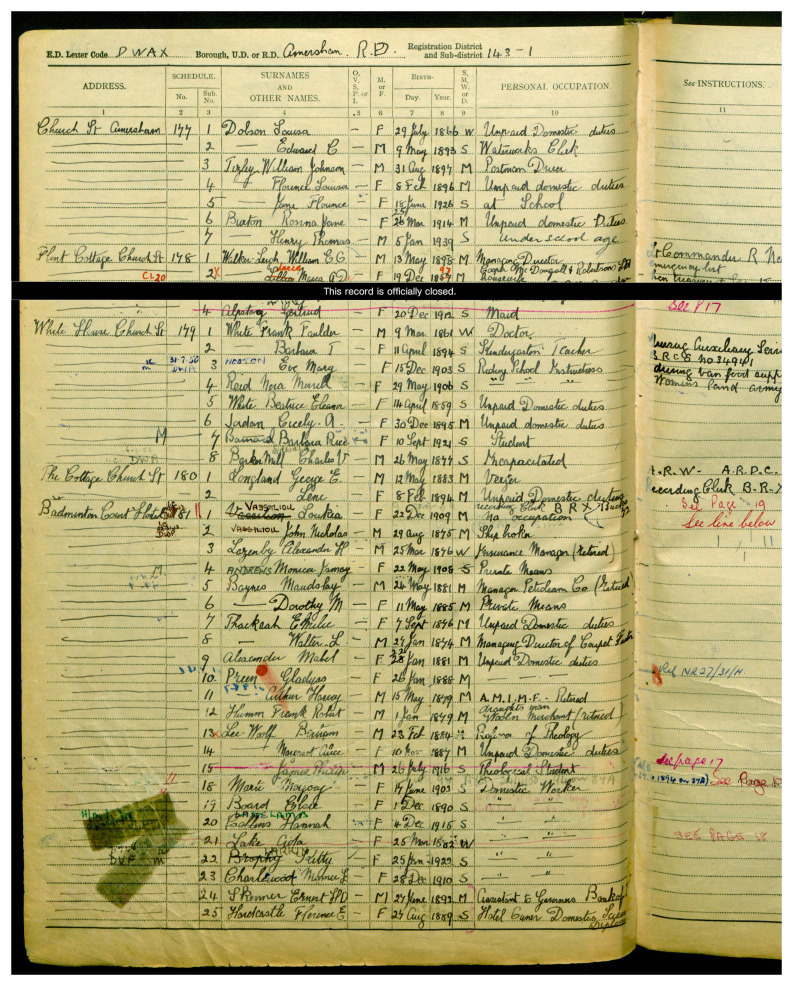
Entry for Loukia Nicolaidou, under her married name Vassiliou, 1939 England and Wales Register. Credit line: with kind permission of the National Archives, Kew, UK, with thanks to Ophélie Siméon. Copyright clearance: The 1939 England and Wales Register is in the public domain according to the 72 Year Rules for Census and Register.

In sum, many of the migrant women who had achieved much in their chosen profession have received little recognition. The inclusion of these women’s tombs in the RMWLE is intended as an act of recovery and memorialisation. This is the case of Loukia Nicolaidou (b. Nicosia, Cyprus 1909–d. 1994, London, UK) who is considered a pioneer of modern art in Cyprus. Her work took her to France (1929–33) and in 1937 she moved to London where she continued to paint and her works became more expressive with a focus on geometrical shapes, influenced by Picasso. However, just as the census of 1939 had recorded her as being of 'no occupation', effectively erasing her identity as a gifted painter, her tomb only lists her married name and no reference to her artistic achievements. (See Figure 2). Studies in memory politics and critical place have heightened the desire for transcultural and inclusive landscapes. However, places that name and tell the stories of migrant women are still rare, as systemic obstacles remain, linked to perceptions of what is acceptable migration for work, as well as what work is acceptable for women.

### 2) Memorializing migrant women in Europe remains a marginal commemoration, tributary of gendered spatial patterns

Mapping the presence or footprint of migrant women in the urban planning of European cities today allows us to approach the resignification of public space. All references to streets, monuments, buildings, etc., dedicated to migrant women in the dataset are geo-referenced so that it is possible to visualise this information in the Map of Women Migrants’ Landmarks and detect spatial patterns and relationships that are elusive to the human eye. When information on professional occupation is added to the spatial coordinates, the results reveal how memory places are configured. From this point of view, the city becomes a cultural construction in which certain elements embody a crucial role over time depending on their spatial location ( Peres *et al*., 2015). These nuances prompt relevant research questions. What values are praised when celebrating migrant women’s identities? And how does this translate into the choice of venue named after them or the choice of monuments chosen to commemorate them?

**Figure 3.**
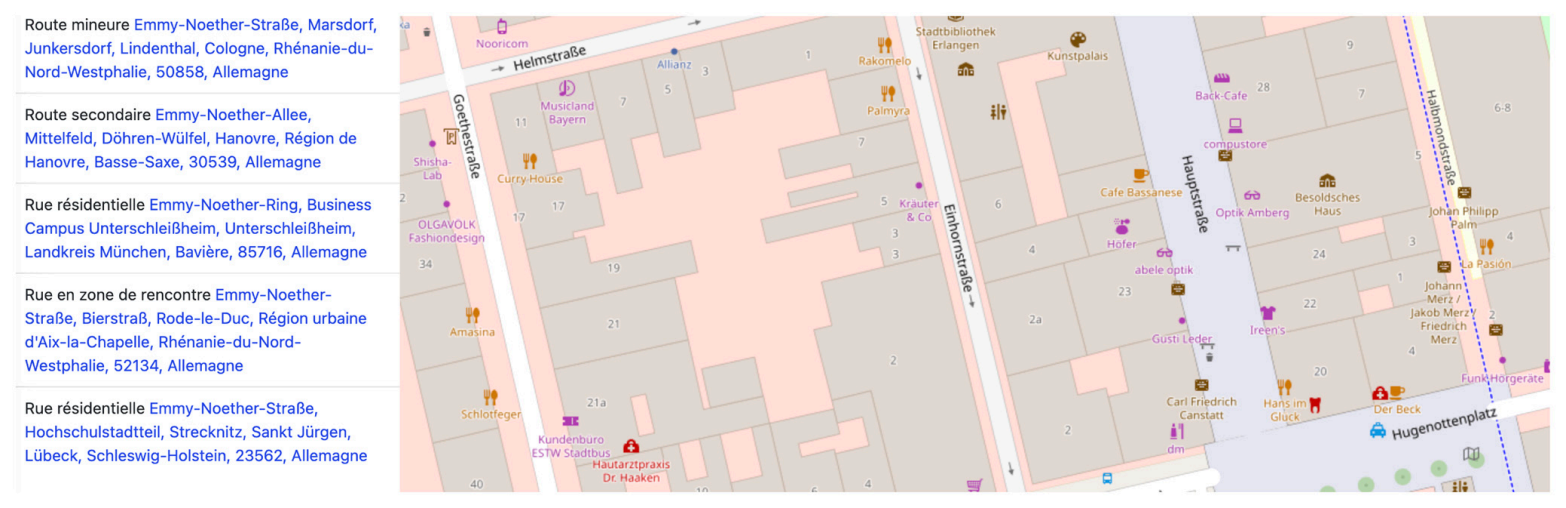
Results for thoroughfares named after Emmy Noether on ©OpenStreetMap. Credit line: ©OpenStreetMap. Copyright clearance: ©OpenStreetMap is an open data, licensed under the Open Data Commons Open Database License (ODbL) by the OpenStreetMap Foundation (OSMF).

On a hopeful note, exceptional examples of how memory policies manifest through the materiality of memorials and place names must also be accounted for. In Spain, the Law 52/2007, commonly known as Historical Memory Law, has favoured replacing the names of public spaces, such as streets or buildings, formerly dedicated to people linked to the Franco regime for women's names ( Romero *et al.*, 2010, 32). However, some studies point to the disproportionate public recognition that exists between men and women, with the latter mostly proposed as new creations rather than replacements, and therefore relegated to the new areas of the city and to the peripheries ( Gutiérrez-Mora & Oto-Peralías, 2022). In this regard, many examples of the marginal distribution of new streets named after women can be found in the RMWLE, such as the street dedicated to the migrant woman and biochemist Gerty Cori – a mere 150 meters of road caught between roundabouts – on a thorough way she shares with another female scientist, Rita Levi-Montalcini, at the technological science park of the city of Getafe. Street named after women migrants in Europe tend to be situated in less busy avenues and boulevards, or in minor ways such as “weg” in Germany, “cale” or “intrarea” in Romania, or “ite” in Serbia. As an example, the following image, a capture from French-language OpenStreetMap, shows all the minor thoroughfares that memorialize the life achievements of Emmy Noether (1882–1935). (See Figure 3). Her work on rings, fields and algebra, and her university teaching for which she migrated to Russia and the USA, was far from minor or secondary but her memorialisation remains confined to marginal spaces.

As explained before, memorialized women tend to overwhelmingly be from the cultural, scientific, cultural and political elite, but even then, their landmarks hold marginal spaces in the cultural landscape. Urban toponomy in most of Europe tends to favour elite professions, and memorialization is often the result of cultural heritage and memory politics choices that are influenced by personalities that are or were deeply embedded in the establishment's networks. This should not of course obscure the fact that this collection of landmarks, if it asserts the potential of migration to be a positive transformation in woman's biographies, has to reckon with the fact that such transformation are context, class and/or network specific, and that the "biographical turning point" ( Hackstaff, 2012) of migration was often negatively disruptive for women unsupported by partners, kinship, communities, networks, financial assets and social capital ( Domecka, 2019). But as we scrolled to geolocate women's monuments and street names, we encountered a pattern of memorialization that hinted at a denser network of visibility in the political landscape of the city. In the light of the above recent sociological research, it was however no surprise that our RMWLE showed that exceptional life accomplishments of the kind that earns a woman public commemoration would frequently include a migration episode. This conclusion is related to the scarcity of empirical research and biographical studies that frame migration as an empowering episode and as a defining life-project rather than a biographical incident or accident ( Boldt, 2012). But if indeed the transformative power of migration on migrant's life is not consistently, or predictably positive, it has been evidenced as frequently transformative when it comes to social mobility and gender perspective ( Bell & Domecka, 2018), for labour opportunities ( Hofmann, 2014) and lifestyle ( Benson & O'Reilly, 2009). Various forms of preserved memories (documents, narratives, memoirs, toponyms, etc.) indeed attest that population migration is at the core of ethno-cultural structuring and social-political organization ( Ristovska-Josifovska, 2020). Our team’s effort has been to read against the grain these notable women's biographies and recover these episodes of migration as decisive assets in these women's social recognition.

**Figure 4.**
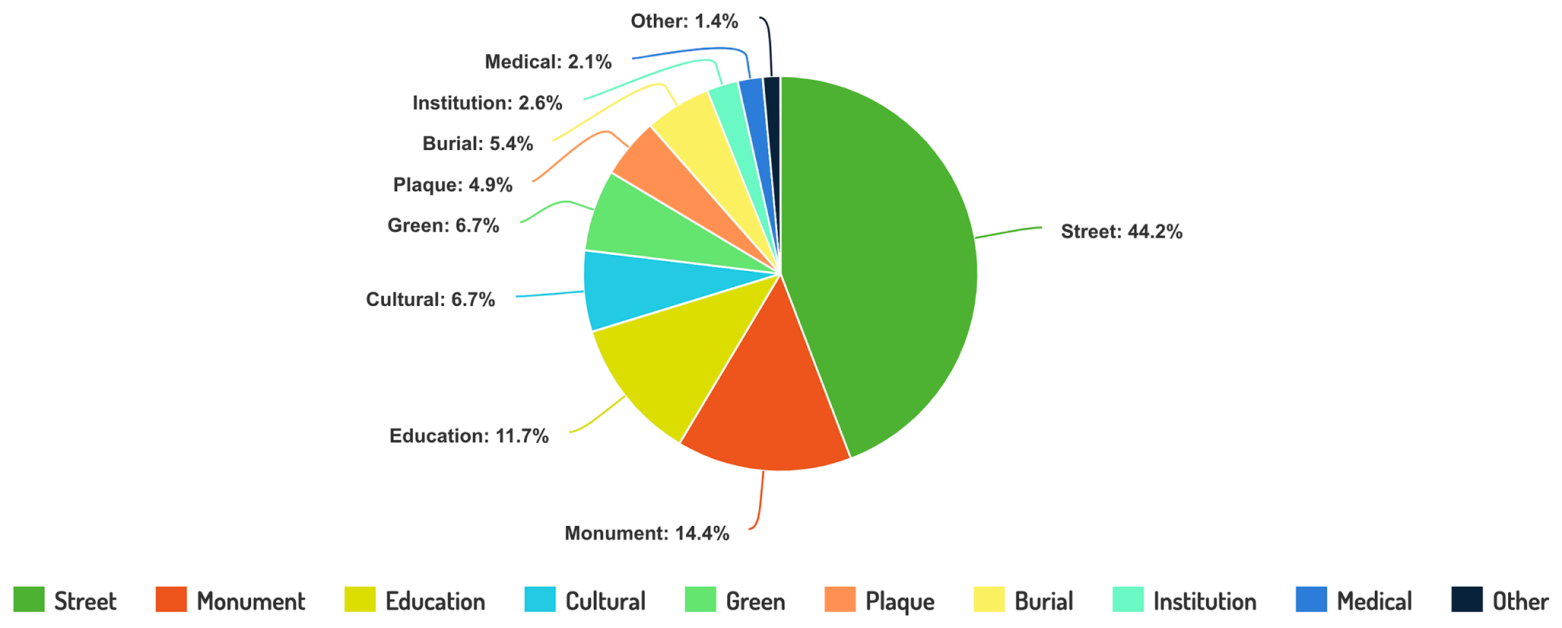
Categories of landmarks in the Register of Migrant Women Landmarks. Credit line: Authors. This circle graph displays the number and relative size of the different categories of toponymic landmarks according to their specificities, based on 1000 entries of the Register of Migrant Women Landmarks. The dataset can be found on NAKALA at https://doi.org/10.34847/nkl.28cf9j6q ( Bakas *et al.*, 2023). It relies on landmark categories that have been devised by the authors according to use and function in the landscape. It shows that memorialisation of migrant women in the European landscape often resorts to street-naming, compared to more costly urban projects such as monument building, or the naming of more prestigious infrastructures, such as hospitals or research institutes.

A review of the 1000 entries classified in categories of landmarks also begins to yield results as to what values are foregrounded when these migrant women are memorialized. The strong presence of the categories “Education Venue (School, University...)” testifies to this ongoing pigeonholing of women’s achievements (See Figure 4).

Debates surrounding monuments and the ideologies they transmit in the last few years have exploded. Monuments within national commemorative spaces often strengthen a specific narrative of the ideal woman ( Stish, 2023). To gain memorialisation, these women had to be found to conform or celebrate specific and stereotypical examples of virtue, patriotism in their lives. Hence their celebration in the urban landscape reinforces ideologies of acceptable feminine identity and creates a misleading legacy for women and their activism in other professions.

The choice of venue or monument chosen to commemorate migrant women is intimately linked with the general perception of women and migrants in terms of their occupational prestige. The RMWLE finds an abundance of 'care and nurture' landmarks, with a high number of results for names of famous migrant women being given to primary schools, kindergarten, and care facilities in conformity with the traditional roles in educating, child-rearing and nursing, career paths that were often the only ones they were allowed to invest in and make their own to become trailblazers. This is the story of Elsie Inglis, whose chosen career path as a surgeon and entrenched enthusiasm for women’s right as a suffragette led her to advocate for the creation of women-staffed medical units during the First World War, a project that emerged as the Scottish Women's Hospitals (SWH), leading her to France, Serbia and Russia despite opposition from the War Office. A nursing home is therefore named after her exceptional achievements in Edinburgh – but so is a kindergarten, early learning and childcare centre.

Data from the RMWLE show that landmarks named after migrant women are not always connected with the occupation that made them famous enough to be commemorated, but rather with notions of care and nature that are stereotypically synonymous with femininity, and have overshadowed the professional woman’s actual achievements. Despite becoming famous for often engaging in stereotypically masculine-coded professions, migrant women’s memorialisation remains connected to care and nurturing. The devaluation of caring roles that are often connected to femininity, is therefore being subtly reinforced by naming schools and caring facilities after famous women whose claim to fame do not rest within these career paths – as can be seen in the landmarks memorialising the Latvian playwright and poet Aspazija (Elza Johanna Emilija Lizete Pliekšāne, 1865–1943) whose career in Lithuania had little to do with the disability care centre named after her in Riga.

The persistent gender-coding of professions and the widely shared perceptions of feminine values have a lasting influence on memorialisation practices – and these practices in turn uphold narrow mindsets that have real-life economic implications. The perception that a job requires stereotypically masculine traits or stereotypically feminine traits is a strong predictor of its being perceived as prestigious and worth of high salary – studies have found that work associated with women is consistently devaluated ( Glick, 1991). Furthermore, the connection of femininity to concepts of primary responsibility for caring remains strong ( Bakas, 2017), and this influences the subsequent constant devaluation of caring work in capitalism ( Federici, 2020). Hence, re-examination of beliefs and narratives embedded in the spaces we inhabit is essential. Commemorations of migrant women in public space gives agency to those outside of established norms by acknowledging their right to the streets and public spaces of the city ( Gill *et al.*, 2020).

Another conclusion drawn from the harvesting of migrant women names given to various infrastructure is the low-cost solution of street naming often chosen by municipalities, which points to a gendered cost of memory and reduced material presence of migrant women in the public space ( Čapská, 2025). Acts of street naming can indeed be characterised as “microtoponymy” since they are a minimal intervention in the historical landscape of the city ( Felecan, 2013). These can appear performative and have a limited impact in terms of storytelling compared to the commissioning of a monument. How involved is the public in reflecting on these women’s biographies? The bestowing of a name to a bus stop or a street is rarely followed by the public having an artistic and cultural experience, both educative and empathetic. Only with added support such as educational tools and visualisation can this microtoponymy initiate the same long-term reflection as in the case of monuments and participatory spaces ( Bancilhon *et al.*, 2021). Recent campaigns to boost the naming of streets after women are encouraging but the public should remain aware of how little money and space is awarded to the memorialisation of women through monuments.

Monument debates in the second decade of the twenty-first century, turning almost entirely on questions of who is represented and by whom, might benefit from considering questions of how the space is experienced, and with what material resources. Art’s mediating power is curtailed if it is destined to simply transmit an official history. A globalised world at the turn of the twenty-first century is the time of the historic monument, recent architectural historians have claimed, thanks to a better understanding not only of the temporary limits of monuments, but also of their strong links to a materialized memory ( Bostenaru Dan *et al*., 2015). The involvement of audiences in the memorial’s physical substance, entering its spaces and otherwise performing acts of commemoration has been central to the most durable memorials of the last half century, and is given a particularly radical turn by artist interested in justice and restitution. The task for theorists of monumentality today, as much as for monument-makers, is to understand how an ethics of care can meet and interact forcefully with a politics of taking responsibility and promoting gender equality ( Widrich, 2020).

## Conclusion

Collecting landmarks memorializing migrant women for the RMWLE has indeed evidences a further minoritization of these women's stories, and the unwillingness to present migrant women as heroines in the urban fabric of the city. However, the register, for all the cultural, political and economic constraints of public memorialization stacked against such migrant women, has enabled us to uncover stories of female migration that were inspiring, often unexpected and subversive in terms of cutting across national master narratives and established gender hierarchies.

By harvesting the data for the RMWLE, we sharpened our understanding of the pitfalls of dominant memorialisation narratives that constrain the stories that are told. We have also reflected on how this register can help combat cultural prejudice by recovering migration episodes, underlining how often these have had a defining impact but how rarely they are underlined in the biographies of famous women.

As we have seen in our second part, there are signs of a growing public desire to acknowledge and honour the historical contribution of migrant women to the history of the countries, places, and communities they belonged to and enriched. The RMWLE offers both historical and more recent instances of their inclusion in the cultural landscape. We hope the RMWLE helps underline the systemic social exclusion in place naming that sees only a selection of career achievements be memorialized, missing out on the inclusive and transcultural richness and dynamism of the stories we uncovered.

As explained in part three, the memorialisation practices at play in most of Europe still gender-codes and marginalizes the achievements of migrant women. We hope the RMWLE helps support calls for more diverse, inventive, and frequent commemoration of migrant women, in the hope that their names and stories will take up more public space and weigh heavier in communal memory.

The dominant narrative in public memorialization continues to be one of marginality, and the selection of these heroines often frames their achievements, despite the odds stacked against them, as exceptional – a reception in the urban landscape that does little to mainstream the recognition of female contribution in general. We learnt that we do more than “live” in places, we are shaped by them and are tasked by the names around us to tell better, more precise, less conventional stories to celebrate these names of migrant women. We wanted to draw attention to the material, ecological and gender-sensitive aspects of commemoration and thus to highlight that they can be shaped in conscious and critical ways. We see our project and dataset as part of a wider effort to encourage the cultivation of more inclusive public memorial landscapes where migrant women are duly represented, and migration experiences recognized.

Ethics and consent - Ethical approval and consent were not required.

## Data Availability

NAKALA : *Memorializing women on the move: A register of migrant women landmarks in Europe*, DOI : 10.34847/nkl.28cf9j6q. ( Bakas *et al.*, 2023) This project contains the following underlying data: DataNote_Final_Dataset_Register of Migrant Women Landmarks in Europe_COSTACTION19112_WeMOV_1000entries 3.xslsx Data are available under the terms of the Creative Commons Attribution 4.0 International ( CC-BY-4.0).

## Notes

1 The dataset is accessible on NAKALA at https://doi.org/10.34847/nkl.28cf9j6q ( Bakas *et al.*, 2023).

2 The Map of Women Migrants' Landmarks in Europe is accessible at https://www.womenonthemove.eu/map-women-migrants-landmarks#leaflet-map-view-pins-map-landmarks-and-exhibitions-block-map-landmarks
