# Supplementary material for: Materiality of Memorialization: Mapping Migrant Women's Landmarks in Europe
Source: Open Res Eur. 2025 Nov 11;4:234. Originally published 2024 Oct 25. [Version 3] doi: 10.12688/openreseurope.18433.3 (PMC11809475; doi:10.12688/openreseurope.18433.3)
Supplement: Peer review history

# Reviewer report: Reviewer response for version 3

**DOI:** 10.21956/openreseurope.23446.r63903

**Published:** 2025-11-24

**Authors:** Minki Sung

**Affiliations:** 1 School of Architecture Planning and Landscape, Newcastle University School of Architecture Planning and Landscape (Ringgold ID: 151479), Newcastle upon Tyne, England, UK

The revised manuscript adequately addresses all of the comments I previously made. I would like to acknowledge the authors' responsiveness and the careful consideration they have given to the issues I raised in my review report. I am pleased to endorse this revised manuscript, and I would like to congratulate the authors on their work.

If any, are all the source data and materials underlying the results available?

Yes

Is the work original in terms of material and argument?

Yes

Is the work clearly and cogently presented?

Yes

Is the argument persuasive and supported by evidence?

Partly

Does the research article contribute to the cultural, historical, social understanding of the field?

Yes

Does it sufficiently engage with relevant methodologies and secondary literature on the topic?

Yes

Reviewer Expertise:

My research explores how history is mobilised through social concepts like heritage, historic urban landscapes, and cultural landscapes. As a heritage and landscape researcher, my interests include heritage values, heritage impacts, conservation planning, and the dynamics of historic and cultural landscapes. I am particularly interested in how the perception and use of history are shaped by society, especially through the lens of heritage and landscape, and how these are influenced by international, national, regional, and local policies.

I confirm that I have read this submission and believe that I have an appropriate level of expertise to confirm that it is of an acceptable scientific standard.

# Reviewer report: Reviewer response for version 3

**DOI:** 10.21956/openreseurope.23446.r63901

**Published:** 2025-11-19

**Authors:** Mihai S. Rusu

**Affiliations:** 1 Lucian Blaga University of Sibiu, Sibiu, Romania

The revised manuscript is a significantly improved version of the initially submitted text. I appreciate the author's throroughness in addressing each of the issues mentioned in the review report. Consequently, I endorse this revised manuscript and congratulate the authors for the article.

If any, are all the source data and materials underlying the results available?

Yes

Is the work original in terms of material and argument?

Yes

Is the work clearly and cogently presented?

Yes

Is the argument persuasive and supported by evidence?

Yes

Does the research article contribute to the cultural, historical, social understanding of the field?

Yes

Does it sufficiently engage with relevant methodologies and secondary literature on the topic?

Partly

Reviewer Expertise:

Gender studies, critical place-name research, sociology of culture, politics of memory.

I confirm that I have read this submission and believe that I have an appropriate level of expertise to confirm that it is of an acceptable scientific standard.

# Reviewer report: Reviewer response for version 2

**DOI:** 10.21956/openreseurope.21337.r51865

**Published:** 2025-03-17

**Authors:** Mihai S. Rusu

**Affiliations:** 1 Lucian Blaga University of Sibiu, Sibiu, Romania

The authors have done a good job in addressing properly all issues pointed out in the review report. As a result, the revised version of the paper is a significantly improved variant of the originally submitted material. Consequently, I endorse an acceptance of this paper in its current form.

If any, are all the source data and materials underlying the results available?

Yes

Is the work original in terms of material and argument?

Yes

Is the work clearly and cogently presented?

Yes

Is the argument persuasive and supported by evidence?

Yes

Does the research article contribute to the cultural, historical, social understanding of the field?

Yes

Does it sufficiently engage with relevant methodologies and secondary literature on the topic?

Partly

Reviewer Expertise:

Gender studies, critical place-name research, sociology of culture, politics of memory.

I confirm that I have read this submission and believe that I have an appropriate level of expertise to confirm that it is of an acceptable scientific standard.

# Reviewer report: Reviewer response for version 2

**DOI:** 10.21956/openreseurope.21337.r51864

**Published:** 2025-03-12

**Authors:** Minki Sung

**Affiliations:** 1 School of Architecture Planning and Landscape, Newcastle University School of Architecture Planning and Landscape (Ringgold ID: 151479), Newcastle upon Tyne, England, UK

This article has significantly improved based on the previous reviewers' feedback. I personally found the revisions in the second manuscript satisfactory. However, I have several suggestions for further enhancing the quality of the work.

 First, by the abstract itself, this article proposes a storytelling approach to challenge dominant cultural narratives and knowledge practices (“This article calls for a storytelling approach, to counter dominant cultural narratives and knowledge practices”). However, introducing this as a research method at the end of the abstract may confuse readers, leaving them uncertain about the overall methodological framework. It is unclear from the abstract whether the study employs a quantitative or qualitative approach (e.g., storytelling). Clarifying this point would help potential readers understand the research methods used to generate the findings. After reviewing the full manuscript, explicitly explaining the mixed-method approach—combining quantitative methods with a storytelling approach at the interpretation stage—would improve clarity and strengthen the methodological foundation.

 Second, power can also be seen as 'power to'. This statement seems to overlook the bottom-up power dynamics that enable low-paid workers to unite and amplify their voices. This 'power to' mechanism challenges the traditional 'power over' model, which assumes that only high-paid, high-status or elite individuals have influence in society, as evidenced by one of your sentences (“Low paid occupations rely on sets of skills that are defined by the labour market as low because they are negatively correlated to one’s amount of influence in society and to the extent of decision taking one is granted.”)

 Finally, there are a few typos or areas to improve clarity.

 What are numerous previous studies? Can you give an example to support your argument? (“Numerous previous studies about women represented in public art have harvested such allegorical representation as denoting female presence in the city landscape nonetheless, but our selection criteria that cross reference womanhood and the experience of migration in the biography of the woman represented made this category of monument irrelevant to the register”)

 Due to? (“The effort of collecting this data is still ongoing, but some areas are better represented as the RMWLE stands due the nature of data collection”). 

 What is power and decision leverage in your research? (“Naming landmarks is an act that necessitates power and decision leverage, and therefore even more susceptible to be a space of systemic patriarchy and mainstream”).

 To whom does the act of outrage refer? Is the term to be interpreted as referring to the general public? (“The fact that Marie Dentière’s plaque, rather than a statue, is neither central nor self-standing, placed as it is at the foot of a male statue, continues therefore to cause outrage”).

If any, are all the source data and materials underlying the results available?

Yes

Is the work original in terms of material and argument?

Yes

Is the work clearly and cogently presented?

Yes

Is the argument persuasive and supported by evidence?

Partly

Does the research article contribute to the cultural, historical, social understanding of the field?

Yes

Does it sufficiently engage with relevant methodologies and secondary literature on the topic?

Yes

Reviewer Expertise:

My research explores how history is mobilised through social concepts like heritage, historic urban landscapes, and cultural landscapes. As a heritage and landscape researcher, my interests include heritage values, heritage impacts, conservation planning, and the dynamics of historic and cultural landscapes. I am particularly interested in how the perception and use of history are shaped by society, especially through the lens of heritage and landscape, and how these are influenced by international, national, regional, and local policies.

I confirm that I have read this submission and believe that I have an appropriate level of expertise to confirm that it is of an acceptable scientific standard, however I have significant reservations, as outlined above.

# Response

**Published:** 2025-10-17

**Authors:** Bénédicte Miyamoto

**Affiliations:** Departement du Monde Anglophone, Universite Sorbonne Nouvelle, Paris, Île-de-France, France

We thank you for this careful review of our article, and the thoughtful suggestions that we hope have now enriched it.

 This new version corrects the abstract section to make the methodological choices clearer, stating we are combining quantitative methods with a storytelling approach at the interpretation stage. In the method and data collection section, we differentiate between surveys that include or exclude allegorical landmarks and have added clear references to both the surveys that exclude or include. This has helped us make clearer the fact that surveys that include allegories are not surveys of women landmarks, but landmarks of femininity, and we thank you for this added clarity. In the Results section, we also give a brief sketch of which jurisdiction or level of governance the decisions to name landmarks generally depend on - and this has also helped make clear (although cases are different European wide) that this generally is divided according to the category of landmarks. This is probably quite useful for those readers that would be specifically interested in our work on landmarks categories. We also nuanced our finding that memorializing migrant women in Europe remains skewed towards the elite - we made it clearer that ESCO categories quantitatively show a higher level of landmarks memorializing elite women, but that ESCO categories are themselves a product of a "power over" analysis. Although we cannot extract from our data of mostly anonymous lower category landmarks much storytelling to support the "power to" narrative, we show now that a sizeable number of the elite landmarks were activists, fighting to give power to minoritized or the downtrodden, and we hope this nuances the hierarchy of the elite's "power over" the other ESCO categories.  We have also corrected typos and updated the reference section.

# Reviewer report: Reviewer response for version 1

**DOI:** 10.21956/openreseurope.19920.r45927

**Published:** 2025-01-03

**Authors:** Andrea Kocsis

**Affiliations:** 1 Design Informatics, University of Edinburgh, Edinburgh, UK

While the project's goal of investigating the connection between gender and migration in memorialscapes deserves praise, the paper would benefit from a few methodological and theoretical suggestions.

 More methodological information would be necessary to support the results, especially if the authors emphasise the role of deep data. The structure, extent, and parameters of the dataset are unclear, and more information on the data collection and management process is required. Particularly, as the analysis points out the presence of bias in the results, knowing the circumstances and limitations of the dataset would be essential. It is hinted that the dataset consists of 1000 entries, and the variables are broadly listed later in the document, however, a more in-depth, explicit, and early explanation of the nature and background of the dataset would be crucial, even if it is linked to the study. For example, the temporal or geographical dimensions of the collected entries are never introduced. It is also unclear what the role of the authors was in the dataset design and data collection. There are also terminological issues, for example, the authors use "database" and "dataset" interchangeably, which suggests confusion about structuring and handling data. For this reason, perhaps omitting the focus on the unstable quantitative angle and highlighting the Register as a valuable but qualitative resource would be a better approach.

 Similarly to the above point, the analysis is not explained. The parameters of the "Qualitative Content Analysis" are not available. Seeing at least the high-level coding categories/themes for CA would be beneficial. However, in its present form, instead of CA, particular case studies were chosen to be investigated after counting the occurrences of some variables in the dataset (e.g., occupation). This approach is also valid, but the project should be explicit about the exact methods. Additionally, the title of the article promises mapping, but more spatial discussions and some GIS analysis would be needed to support the argument. For example, the observation about the landmarks' often peripheral locations is fascinating but should be supported by analysis. 

 Regarding theory, the article should consider three points. The first is the temporal dimensions of the collected landmarks. Urban memory is transient, and -  depending on their geographical location and national/governmental backgrounds - memorials are constantly moved, removed, modified, and reinstalled. How this is accounted for in the analysis or recorded in the dataset is unclear. Secondly, in connection with the above, introducing and evaluating the landmarks' geographical distribution would be crucial. For example, not the same memory political approach applies to the memorialscape of Central Europe and the UK. Hence, handling them together might distort the results. Knowing the geographical distribution would be essential as the changing Authorised Heritage Discourse (cf. Smith 2006) from state to state, municipality to municipality, and regime to regime defines the commemorative landscape. Finally, the historical context of migration should matter. For example, the memory political reception of migration in the case of a 16th-century foreign princess and a Jewish nurse escaping the Nazi regime is different. However, as the temporality of the dataset is not explained, the paper does not distinguish historical contexts. 

 Considering the findings, the study would benefit from reflecting on the possibility that commemoration is generally the business of the elit, regardless of gender or settlement status. Furthermore, the study points out an interesting hypothesis about the Register demonstrating that "exceptional accomplishments of the kind that earns a woman public commemoration would frequently include a migration episode." However, the abovementioned lack of detailed methodology makes it unclear what supports this conclusion. The intersectional focus is the strength of the project, but the dataset on its own might not be enough to draw this inference without tautology. To further prove the relationship between migration and gender in memorialscapes, it would be essential to cross-reference the results with data on landmarks dedicated to non-migrant women and/or migrant men. 

 The argument is strongest and most supported regarding the evaluation of the values and stereotypes for which women are commemorated. However, as mentioned above, it is unclear how specific it is to migrant women.

 Overall, to achieve the important and timely aim of the project, namely highlighting the connection between historical migration and gender in the urban landscape, the study would benefit from either clarifying the methodological, quantitative, and data angles of the paper or explicitly adopting the narrative storytelling approach. As Benjamin M. Schmidt recently pointed out, narrative storytelling is a welcome and perfectly valid angle of digital history, and emphasising the strength of historical narration might be a better way than imperfectly quantifying the sources (cf. Schmidt 2024)- **Schmidt, B. M. (2024). Two Volumes. The Lessons of Time on the Cross. In: Tilton, L., Mimno, D., & Johnson, J. M. (eds). Computational humanities. University of Minnesota Press.161-176.**

 ISBN: 9781517915988, ISBN-13 ‏ : ‎ 978-1517915971

 .

If any, are all the source data and materials underlying the results available?

Yes

Is the work original in terms of material and argument?

Yes

Is the work clearly and cogently presented?

Partly

Is the argument persuasive and supported by evidence?

Partly

Does the research article contribute to the cultural, historical, social understanding of the field?

Partly

Does it sufficiently engage with relevant methodologies and secondary literature on the topic?

Partly

Reviewer Expertise:

My research interest is in the intersection of Heritage Studies and Data Science, with a special focus on urban landscapes. I'm an interdisciplinary scholar with a background in Memory Studies, Heritage Studies, Urban History, Computational Humanities, Digital History, and Data Science.

I confirm that I have read this submission and believe that I have an appropriate level of expertise to confirm that it is of an acceptable scientific standard, however I have significant reservations, as outlined above.

## References

[1] : Uses of Heritage.2006; 10.4324/9780203602263 10.4324/9780203602263

# Response

**Published:** 2025-02-17

**Authors:** Bénédicte Miyamoto

**Affiliations:** Departement du Monde Anglophone, Universite Sorbonne Nouvelle, Paris, Île-de-France, France

As the project is winding down and coming to a close (March 2025) we are grateful for the through methodology advice that you have offered. Your just remarks cannot be all addressed due to the constraints of the project and the realities of collaborative work, but we have implemented the following.

 We thank you for reminding us that simply linking the Data Note and Dataset is insufficient to support the article, and that the structure, extent, and parameters of the dataset must be made explicit in the body of the article. With help from yours and other reviewers’ reports, we have made changes to:  

1. **The methodology section**:

We have specified the temporal and geographical dimensions of the collected entries as follows and addressed the question of erased or displaced landmarks in our register.   [ “Our network linked 52 scholars and students, listed as having contributed to the collection of data for the RMWLE in the Data Note ( Ojala-Fulwood et al., 2024). These researchers coming from 41 different countries built a dataset covering 41 countries (Albania, Austria, Belgium, Bosnia and Herzegovina, Bulgaria, Croatia, Cyprus, Czech Republic, Denmark, Estonia, Finland, France, Georgia, Germany, Greece, Hungary, Iceland, Ireland, Israel, Italy, Latvia, Lithuania, Luxembourg, Malta, Moldova, Montenegro, Netherlands, North Macedonia, Norway, Poland, Portugal, Romania, Serbia, Slovakia, Slovenia, Spain, Sweden, Switzerland, Turkey, Ukraine, United-Kingdom). We eschewed historical boundaries in our collection, which currently spans from the 9 ^th^ century to the present, to build a picture of what, as of now, is present in the European architectural memorial landscape (rather than a picture of landmarks erected or named since a specific date; or landmarks referring to women active during a specific period). The main goal of the article was to be able to give current quantitative data to highlight the dearth of figures in the memorial landscape that intersect both migration history and the identity of women. We did not allow for landmarks that have disappeared or been replaced, and if such landmarks from older databases or historical documents appeared at the collection stage, the need for currently valid geo-coordinates to be registered in our dataset enabled us to weed out erased or displaced landmarks.”]  

1. **Part 1: Memorializing migrant women in Europe remains skewed towards the elite **

   We thank you for the reminder that that commemoration is generally the business of the elite, regardless of gender or settlement status, which we have now underlined in this Part 1 intro, and we have stressed that  no studies to this day has analyzed the distribution of non-migrant male or female landmarks in terms of social prestige linked to occupation, skills and competences. This has therefore qualified the conclusions we can as yet infer from the register, given the lack of comparative studies.   We thank you also for the validation of the labour categories we built into the register’s design, on which we based our evaluation of the values and stereotypes for which women are commemorated, which you underlined was the strongest point of the article. We strove to reinforce this merit of the article by delving more deeply into the exact photo fit of the memorialized professional women (Esco Category 2, the largest category memorialized) by visualizing a more in-depth break down of the skills and competences that were deemed worthy of memorialization. We recoded the Esco category column of these category 2 women to add a more granular level of occupation skills and competences, which can be found in the newly submitted *Figure 2 – Professional Migrant Women in the Register of Migrant Women Landmarks*, which uses three-digit ESCO categories instead of 1-digit. We added a discussion of this new figure to the text.

    

1. **Part 2: Memorializing migrant women in Europe remains a marginal commemoration, tributary of gendered spatial patterns**

  WE thank you for underlining that the study points out an interesting hypothesis about the Register demonstrating that "exceptional accomplishments of the kind that earns a woman public commemoration would frequently include a migration episode." We agree however, that lack of comparable studies about non-migrant women or non-migrant men make these a hypothesis as of yet unproven. We have amended the text as follows: “We encountered a pattern of memorialization that hinted at a denser network of visibility in the political landscape of the city. In the light of the above recent sociological research, we suspect that our intersectional RMWLE could be used to posit that exceptional life accomplishments of the kind that earns a woman public commemoration would frequently include a migration episode. Such a conclusion cannot be drawn yet, due to the absence of survey studies on non-migrant women (or male) landmarks, however. There is furthermore yet too few empirical research and biographical studies that frame migration as an empowering episode and as a defining life-project rather than a biographical incident or accident ( Boldt, 2012). (…) Our team’s effort has been to read against the grain these notable women's biographies and recover these episodes of migration. We hope further comparative studies and efforts of case studies will continue to investigate these migration episodes as decisive assets in these women's social recognition.”    

1. **Addendum on Case studies**

  We have stressed that the quantitative register is unstable, as you have rightly pointed out, and remind the reader that “the available empirical material of migrant women landmarks “in situ” is subject to change as the memorial landscape evolves, and our research has been deliberately conceived as open-ended and open access”. To this end, we have both corrected our mentions of Case Studies in the Methodology sections and our references to case studies in the body of the text (our examples bring alive the register to the reader but we point out that they are not studies in the methodological sense). We devised an addendum at the end of the article calling for the development of local, municipal and nation level Case Studies analysis using the register. In this addendum, we stressed that case studies concentrating on geographical or temporal distributions would be essential to reflect the changing “authorized heritage discourse” from state to state, municipality to municipality, and regime to regime, and how this defines the commemorative landscape (we reference the work of Smith 2006). In this regard, we have also introduced specific research to support points in the body of the text to highlight what local case studies (for example the research of Rusu on gendered street naming in post-communist Romania, 2024; or the research of Sèbe on racial patterns in post-colonial French DOM-TOM, 2020)  or municipal case studies (we have added reference to the research of Novas Ferradás on Santiago de Compostela City’s Urban Toponymy, 2018) can bring as for nuance and historically specific patterns. We also stressed that quantitative methods are appropriate for our subject (we reference the work of Krippendorff 2019) but benefit from being doubled by storytelling at the interpretation stage, as referenced in the methodology section (where we referenced Parry, 2020). 

 We are grateful for the methodological backing you offered about Case Studies and storytelling approaches and have built on them into the addendum (notably relying on Hamilton, Howard, and Pick, 2008’s call to action).

# Reviewer report: Reviewer response for version 1

**DOI:** 10.21956/openreseurope.19920.r48015

**Published:** 2024-12-18

**Authors:** Mihai S. Rusu

**Affiliations:** 1 Lucian Blaga University of Sibiu, Sibiu, Romania

The article provides a timely analysis of a scarcely research topic, that is, the memorial artefacts dedicated to migrant women across Europe. This is a timely piece due to a couple of reasons: first, research on women’s invisibility in the symbolics of public place is gaining traction, and so are the claims to address it coming from feminist movements and other civic organizations; secondly, the article examines the memorialisation of migrant women, where migration adds a second layer of invisibility to that of gender. The article is based on an impressive dataset including 1,000 memorial artefacts dedicated to migrant women throughout Europe, which provides the empirical basis for developing enlightening statistical analyses that shed light on who, how, and where gets to be commemorated within this category. While the dataset is impressive and the analyses are revealing, the article could nevertheless benefit from several revisions, as pointed out below:

1. **Introduction:** the authors write that the Register of Migrant Women Landmarks in Europe register “not only odonyms, (street names) but a vast array of urban toponyms (from monuments to such varied urban infrastructure as nurseries and hospitals)”. Monuments are not toponyms and therefore the phrase should be rewritten so as monuments not to be enumerated as a particular case of toponyms.
2. **Introduction:** “the abundance and prominence of landmarks devoted to men compared to women is a subject well-covered in memorialisation studies”. While this is certainly true, the authors could specify several examples of recent research that highlight this gender disparity in the politics of commemoration.
3. **Introduction:** “these cross-border celebrations remain rare”. Is “celebration” the most appropriate word here? I would suggest considering using commemoration instead.
4. **Methods and data collection: **the authors write that they use “Qualitative Content Analysis” and “Case Study” methods. First and foremost, the study is based on a quantitative approach that allows authors to produce statistical analyses. As such, the method should be “quantitative content analysis” (see, in this regard, Klaus Krippendorff’s “Content Analysis: An Introduction to Its Methodology”). Secondly, I am not convinced that the reference to “case study method” is appropriate either. The fact that the authors pick up some particular migrant women and briefly discuss their biographies and how they were commemorated is not enough to characterize the approach as a “case study” research (see, in this regard, Robert K. Yin’s “Case Study Research: Design and Methods”).
5. **Methods and data collection: **“The dataset falls under the concept of Deep Data versus Big Data”. This sentence is not clear. Please revise to “Deep Data as opposed to Big Data”.
6. “The collection of data was based on voluntary work of scholars and students from over 40 different European countries”. I suggest specifying the exact number of countries.
7. “…a strict national border criterion cannot not be used consistently”: please revise the double negative so as to make the sentence clear.
8. “These are consistent with similar research conducted on European toponyms”: see, in this regard, the research done on the national street nomenclature of Romania – Rusu, 2024 (Ref - 1), https://www.sciencedirect.com/science/article/pii/S0277539524000475.
9. Figure 1: For better clarity, I suggest adding the number/frequency or the percentages on top of each bar representing women’s occupations.
10. Figure 3: This figure is not the best to emphasize that street named after women tend to be located towards the outskirts of towns and/or to be smaller in size/length. I suggest authors to come up with a better visualisation of this important pattern.
11. Figure 4: For improving the comparability of the results, I would suggest authors to change the pie chart they are using with a bar chart, with percentages mentioned on top of each bar.

If any, are all the source data and materials underlying the results available?

Yes

Is the work original in terms of material and argument?

Yes

Is the work clearly and cogently presented?

Yes

Is the argument persuasive and supported by evidence?

Yes

Does the research article contribute to the cultural, historical, social understanding of the field?

Yes

Does it sufficiently engage with relevant methodologies and secondary literature on the topic?

Partly

Reviewer Expertise:

Gender studies, critical place-name research, sociology of culture, politics of memory.

I confirm that I have read this submission and believe that I have an appropriate level of expertise to confirm that it is of an acceptable scientific standard, however I have significant reservations, as outlined above.

## References

[1] : “No woman's land?” The gendered patterning of urban street names in Romania. *Women's Studies International Forum* .2024;104: 10.1016/j.wsif.2024.102909 10.1016/j.wsif.2024.102909

# Response

**Published:** 2025-02-17

**Authors:** Bénédicte Miyamoto

**Affiliations:** Departement du Monde Anglophone, Universite Sorbonne Nouvelle, Paris, Île-de-France, France

As our project approaches its conclusion in March 2025, we would like to express our gratitude for your methodological guidance, especially in strengthening our Quantitative Content Analysis approach, and we appreciate your thorough review of our text. Your feedback has been invaluable in refining our second version of the article, as follows:  

***Introduction:*** *Monuments are not toponyms*:

**Response: **we thank you for this correction, which we effected in the introduction and abstract. We also clarified in the corpus of the text by fleshing out, when necessary, the use of “toponyms” to read as “monuments and toponyms” or more generally “landmarks”.  

***Introduction:*** *the authors could specify several examples of recent research that highlight this gender disparity in the politics of commemoration.*

**Response: **We have included references to research highlighting the continued hold of the “great men of history” narrative. We added to the introduction and to the second paragraph of the first part the following information: a reminder to our reader that the European architectural landscape we are dealing with is particularly androcentric, (Novas Ferradás 2018), and that the European Data Journalism Network finds that just 6 out of the 100 most popular figures in the street names of 15 European capitals are women (https://mappingdiversity.eu/). We for example added that “progress is slow, and researchers underline that attempts to resist the lack of diversity in public art often falls short of addressing the gender imbalance, continue to obscure women or uphold gender stereotypes, as recent surveys of anticolonial and abolitionist statues in French Caribbean islands has shown (Sèbe 2020, McGinnis 2022). All in all, according to Maren Reeder’s study of monuments to notable women, little has changed since Nuala Johnson’s study that showed that European debates surrounding national identities were articulated in the public life of cities, great and small, around gendered statues that reveal a masculine national imagined community, especially in Eastern European urban areas (Johnson 1994; Reeder 2018)”.  

***Introduction:*** *“these cross-border celebrations remain rare”. Is “celebration” the most appropriate word here? I would suggest considering using commemoration instead :*

**Response: **Indeed, we have changed this term in the introduction, and generalized the use of commemoration in the body of the article, adding at one point the caveat emptor that many of our landmarks memorialize difficult and traumatic events that are no cause for celebration – and that memorialization does not always come with a blanket approval of a memorialized person’s biography.  We thank you for this important nuance.  

***Methods and Data Collection:***

***Response:*** We thank you for this important clarification and method strengthening references. We have updated our methodology section by explaining that our study is based on a quantitative content analysis that allows authors to produce statistical analyses, “an unobtrusive technique that allows researchers to analyze relatively unstructured data in view of the meanings, symbolic qualities, expressive contents they have and of the communicative roles they play in the lives of the data's sources” (Krippendorff 2019, 51).  We for example added the following references to show the appropriateness of quantitative content analysis to our subject since quantitative content analysis, specifically when examining visual materials “helps us to examine important questions of ideological influence and the ways in which particular versions of reality are constructed and fashioned over time and across a variety of media (…) in order to both devise meaningful categories in the research design and to enable interpretation of why such resulting frequencies or omissions are culturally or politically significant” (Parry 2020, 352). We have reminded the reader that we hope this will in turn “enable "Case Studies" from researchers using our database, through in-depth examination of specific scenarios, such as the treatment of migrant women in a particular European country or professional sector, to highlight locally specific or historically specific cases of memorialization of migrant women which resist the homogeneity of a Europe-wide dataset, for example in settings of decommunization and ideological struggles with nationalism, or in cities marked by nineteenth-century bourgeois high art values enshrined by logics of preservation.” We have further clarified in an addendum that our approach did not rely on case studies – although we do ponder on specific examples sometimes – and we have made an express call for our quantitative survey to be strengthened by historical case studies researchers who would use our dataset.   As suggested, we have revised “Deep Data versus Big Data” to “Deep Data as opposed to Big Data”, and specified the exact number of countries our researchers came from, as well as corrected the typos mentioned.  

**Figures:**

**Response: **We have made further changes to the Figures, opting for a bar graph presentation for both Figure 1 and 4 (now 5) as suggested, and adding a Figure 2 to break down the composition of the category “2 – Professionals” that represents most occurrences, and for which a more detailed and granular view was required. We have also added visible tags of results to each graph as advised.   We would like, in conclusion, to thank you for the additional bibliographical references you have provided which significantly strengthened the article (Rusu 2024; Yin 2003; Krippendorf 2019).

# Reviewer report: Reviewer response for version 1

**DOI:** 10.21956/openreseurope.19920.r48019

**Published:** 2024-12-11

**Authors:** Minki Sung

**Affiliations:** 1 School of Architecture Planning and Landscape, Newcastle University School of Architecture Planning and Landscape (Ringgold ID: 151479), Newcastle upon Tyne, England, UK

This research examines how migrant women are marginalised in the built environment and how unequal heritage mobilisation contributes to ongoing normative memorialisation practices. The necessity and theoretical underpinnings of the study are well articulated, as are its analytical findings. However, there are several suggestions for improving the project in the future.

 First, municipalities have considerable power in determining which aspects of history are mobilised and celebrated within the built environment. Examining the specific political and power dynamics behind decisions such as naming streets or erecting statues - and understanding the motivations behind these decisions - could provide deeper insights into migrant women's heritage mobilisation.

 Second, providing additional details about the dataset used for analysis could enhance the clarity and impact of the study. For example, how many women's stories were included in the analysis? What criteria were used for inclusion or exclusion? Without clear engagement with the dataset, the research process may appear somewhat unclear. In addition, given the range of qualitative and possibly quantitative data used, including visualisations of the data collected might help readers to better grasp the breadth and depth of the analysis.

 Third, a more in-depth exploration of the intersection of migrant and gender identities could strengthen the findings. While the research focuses on both 'women' and 'migrants', the link between these two identities could be further developed. For example, a comparative analysis of migrant men and women could shed light on the gendered implications of historical memorialisation and mobilisation in the built environment. This deeper examination of the interplay between migrant status and gender could provide valuable insights.

 In other words, the research focus seems to overlap at times, raising the question of whether the primary lens is 'migrants' or 'women'. Considering the intersectionality of these identities, as well as other factors such as race or class, may be crucial. A sharper focus on the experiences of migrant women, particularly through the lens of race, may reveal additional layers of meaning. For example, the study touches briefly on elite women, but this aspect could be further developed by considering an additional dimension of race and class.

If any, are all the source data and materials underlying the results available?

Yes

Is the work original in terms of material and argument?

Yes

Is the work clearly and cogently presented?

Yes

Is the argument persuasive and supported by evidence?

Partly

Does the research article contribute to the cultural, historical, social understanding of the field?

Yes

Does it sufficiently engage with relevant methodologies and secondary literature on the topic?

Yes

Reviewer Expertise:

My research focuses primarily on how history is mobilised in different ways around social concepts such as heritage, historic landscape and cultural landscape. I have a multi-disciplinary academic and professional background in history, archaeology, archival studies and conservation planning. I uses a comparative, interdisciplinary and multi-method approach, combining a range of research methods including participant observation, in-depth qualitative interviews, archival research, thematic analysis, survey techniques and statistical modelling. His quantitative research uses both primary data collected through small and medium-sized online surveys and secondary data from high-quality national databases.

I confirm that I have read this submission and believe that I have an appropriate level of expertise to confirm that it is of an acceptable scientific standard, however I have significant reservations, as outlined above.

# Response

**Published:** 2025-02-17

**Authors:** Bénédicte Miyamoto

**Affiliations:** Departement du Monde Anglophone, Universite Sorbonne Nouvelle, Paris, Île-de-France, France

As our project approaches its conclusion in March 2025, we would like to express our gratitude for your thorough methodological guidance. Your feedback has been invaluable in refining our second version of the article. While we acknowledge that not all suggestions could be fully implemented due to project constraints and the complexities of collaborative research, we are thankful for your several suggestions for improving the project in the future, and we strove to signal these in the article, for those who we hope will be using and building the register in the future.  

 We have also incorporated the following revisions to strengthen our work, with help from yours and other reviewers’ concurring reports:  

**1) The methodology section**:

    We have specified the temporal and geographical dimensions of the collected entries, and provided additional details about the dataset used for analysis. We thank you for reminding us that simply linking the Data Note and Dataset is insufficient to support the article, and that the structure, extent, and parameters of the dataset must be made explicit in the methodology section of the article. We have added for example, what criteria were used for inclusion or exclusion from the Register of Migrant Women’s Landmarks in Europe and specified what counted as “migratory experience” in the biographies of these 1000 women for them to be entered into the dataset.  

**2) Part 1: Memorializing migrant women in Europe remains skewed towards the elite **  

    We thank you also for your comment on how the study touches on elite women, but could be further developed by considering an additional dimension of class, for example. Our addition of ESCO categories to the dataset, on which we based our evaluation of the values and stereotypes for which women are commemorated, was indeed lacking in a more in-depth analysis of the class status and skills recognition of the majority of the women memorialized – the professional women.  We recoded the Esco category column of these ESCO category 2 women to add a more granular level of occupation skills and competences, which can be found in the newly submitted *Figure 2 – Professional Migrant Women in the Register of Migrant Women Landmarks*, which uses three-digit ESCO categories instead of 1-digit. We added a discussion of this new figure to the text.   We thank you also for your comment on how the study touches on elite women, but could be further developed by considering an additional dimension of race. We have added comparison to masculinity and race studies that rely on landmark surveys (Johnson, N. Cast in Stone: Monuments, Geography, and Nationalism; McGinnis, L. Memorializing Masculinity? Gendering the Iconography of French Colonialism and Anticolonial Resistance in Martinique and Guadeloupe, 2022; Sèbe, B : Colonial heroes, 2020). We have also made clear one of the blind spot of the article, since no studies to this day has analyzed the distribution of non-migrant man or women landmarks in terms of social prestige linked to occupation, skills and competences. We reminded the reader that we cannot compare the specificities of our intersectional dataset of migrant women landmarks to other groups until this work has been done.  

**3) Addendum**

    We devised an addendum at the end of the article calling for the development of local, municipal and nation level Case Studies analysis using the register. As you rightly pointed out, municipalities have considerable power in determining which aspects of history are mobilised and celebrated within the built environment. In this addendum, we also stressed that case studies concentrating on geographical or temporal distributions would be essential to reflect the changing “authorized heritage discourse” from state to state, municipality to municipality, and regime to regime, and how this defines the commemorative landscape (Smith 2006). We also stressed that quantitative methods are appropriate for our subject (Krippendorff 2019) but benefit from being doubled by storytelling at the interpretation stage, and at a more local level, as referenced in the methodology section (Parry, 2020) and the addendum (Hamilton, Howard, and Pick, 2008, i).

# Reviewer report: Reviewer response for version 1

**DOI:** 10.21956/openreseurope.19920.r45598

**Published:** 2024-11-06

**Authors:** Eva Johanna Holmberg

**Affiliations:** 1 University of Helsinki, Helsinki, Finland

This research article does important cultural work in unveiling the many ways migrant and mobile women's memorialisation has been skewed and marginalised in collective urban acts of commemoration (by naming streets and urban areas, sculptures, and other material means). It also produces knowledge about what types of mobile and migratory women's life stories have been recorded (which has been skewed towards educated urban elites and women of higher social classes and political positions). When developing the project in the future, the authors might want to put more emphasis on how a wider set of women's stories might be preserved, also outside the classic urban commemorative practices, paying attention to an even wider set of intersectional inequalities, the fluidity and mixing of gender and race, and the changing practices and actions of mobility and migration within an individual life course - whether trans-women might be included in the categories of womanhood more explicitly, and whether mixed-race backgrounds and a great variety of refugee and forced migration would be included in these identity categories that are commemorated in the future, and what is the role of 'non-European' or 2nd generation migrant women amongst 'women on the move'? Do migrant women always have to become 'heroines' or is that one form of assigning them with additional requirements to fit in? These are just some examples of the type of questions I would pose in an informal dialogue with the project participants but might be worth pondering more in the future if and when the project expands.

If any, are all the source data and materials underlying the results available?

Yes

Is the work original in terms of material and argument?

Yes

Is the work clearly and cogently presented?

Yes

Is the argument persuasive and supported by evidence?

Yes

Does the research article contribute to the cultural, historical, social understanding of the field?

Yes

Does it sufficiently engage with relevant methodologies and secondary literature on the topic?

Yes

Reviewer Expertise:

Cultural history, history of mobility, cultural encounter, travel writing studies, life writing studies

I confirm that I have read this submission and believe that I have an appropriate level of expertise to confirm that it is of an acceptable scientific standard.
